# Angiographic Anatomy of the Left Coronary Veins: Beyond Conventional Cardiac Resynchronization Therapy

**DOI:** 10.1007/s11886-025-02204-z

**Published:** 2025-02-19

**Authors:** Iulia-Georgiana Zehan, Csilla-Andrea Eötvös, Mădălina Patricia Moldovan, Mihai-Gabriel Andrei, Călina-Patricia Șchiop-Țentea, Roxana Daiana Lazar, Adriana Daniela Sârb, Giorgia Coșeriu, Teodora Avram, Adela Șerban, Gabriel Gușetu, Larisa Diana Mocan-Hognogi, Roxana Chiorescu, Sorin Pop, Edwin Kevin Heist, Dan Blendea

**Affiliations:** 1 Iuliu Hatieganu University of Medicine and Pharmacy, Niculae Stancioiu Heart Institute, Cluj-Napoca, Romania; 2 Department of Cardiology, Rehabilitation Hospital, Iuliu Hațieganu University of Medicine and Pharmacy, Cluj-Napoca, Romania; 3 Department of Medicine, Cluj County Emergency Hospital, Iuliu Hațieganu University of Medicine and Pharmacy, Cluj-Napoca, Romania; 4 Demoulas Center for Cardiac Arrhythmias, Massachusetts General Hospital, Harvard Medical School, Boston, Massachusetts USA

**Keywords:** Coronary sinus, Coronary venous anatomy, Coronary sinus angiogram, Cardiac veins, Left ventricular summit veins, Left atrial veins

## Abstract

**Purpose of Review:**

This review aims to synthesize current knowledge on the angiographic anatomy of the coronary sinus and its tributaries veins, with focus on venous branches other than classical ones used in cardiac resynchronization therapy. It also presents common anatomical aspects that could impact the clinical outcome.

**Recent Findings:**

Recent advancements in the electrophysiology field, like epicardial arrhythmia mapping and ablation through coronary sinus or cardiac pacing from atypical veins requires a detailed angiographic assessment of cardiac veins. There is an increased interest for the veins coursing in the left ventricular summit (LVS) area and could potentially provide a pathway to reach the LVS arrhythmogenic foci. However, there is no consensus regarding the nomenclature and classification of these veins.

**Summary:**

This review could offer a better understanding of the coronary sinus and its tributary veins distribution, dimensions and relationship with nearby structures that could help the development of new ablation and pacing tools and strategies, with higher success rates.

## Introduction

The first information about cardiac veins dates to the Renaissance period, when Leonardo da Vinci showed interest in human anatomy, particularly in the cardiovascular system. His valuable drawings, which were based on necropsy studies, became the cornerstone of modern anatomy for future generations of anatomists [1, 2].

In the eighteenth century, new insights into the venous circulation of the heart emerged. The French anatomist Raymond de Vieussens demonstrated in his work “Nouvelles Decouvertes sur le Coeur” (1706) that there is a communication between the coronary arteries and cardiac chambers. His assumption was based on an experiment that involved the ligation of the superior and inferior vena cava and pulmonary veins and the injection of saffron tincture into the left coronary artery. He observed the presence of yellow dye not only in the right atrium, which was supposed to be drained trough the coronary sinus (CS), but also in the left and right ventricles. This finding led to the idea that there are small communicating veins inside the myocardial walls, which he named “ducti carnosi” [3]. A few years later, the German anatomist Adam Christian Thebesius described and classified these veins by size and number in his doctoral dissertation “Dissertatio medica De circulo sanguinis in Corde” (1708) as “thebesian veins” or “venae cordis minimae” [2, 4].

The coronary venous (CV) system plays a crucial physiological role in draining deoxygenated blood from the cardiac structures. Additionally, it serves as an access path for various electrophysiological diagnostic and treatment procedures. The angiographic description of the anatomy of the CV system is of great importance for the successful implementation of ablation therapy, arrhythmia mapping, cardiac resynchronization theraphy (CRT) as well as new percutaneous CS interventions [5].

## Classification of the Coronary Venous Tree

Since the first description of the venous vasculature of the heart until now, several classification systems have been proposed. Taking into consideration their localization and drainage site, three groups of veins can be individualized. The first category consists of the CS and its branches, responsible for draining most of the myocardium. The second group is formed by the veins collecting the blood from the anterior part of the heart, including the right ventricle, and opening into the right atrium. The third group is represented by the small, intramural vessels, known as Thebesian vessels, that open directly into any chamber of the heart [2, 6, 7].

A recent classification divides the cardiac venous system into only two categories: the “*greater venous system”*, consisting of the veins located in the outer layer of the myocardium (CS with its tributary veins and anterior cardiac veins) and the *“lesser venous vasculature”,* that is the Thebesian system [2].

From the point of view of the electrophysiologist, another classification method based on the drainage region of the cardiac veins, the so-called *“segmental classification”,* was proposed to help position the left ventricular pacing lead for CRT. This classification refers to the veins of the left ventricle (LV) and divides them along the horizontal and vertical axes, resulting in 9 LV segments [6]. Along the longitudinal axis, the second-order tributaries are classified as belonging to the basal, mid, or apical region. Circumferentially, each region has anterior, lateral, and posterior segments. It helps to describe with more precision the epicardial course of the cardiac veins [6].

**Fig. 1:**
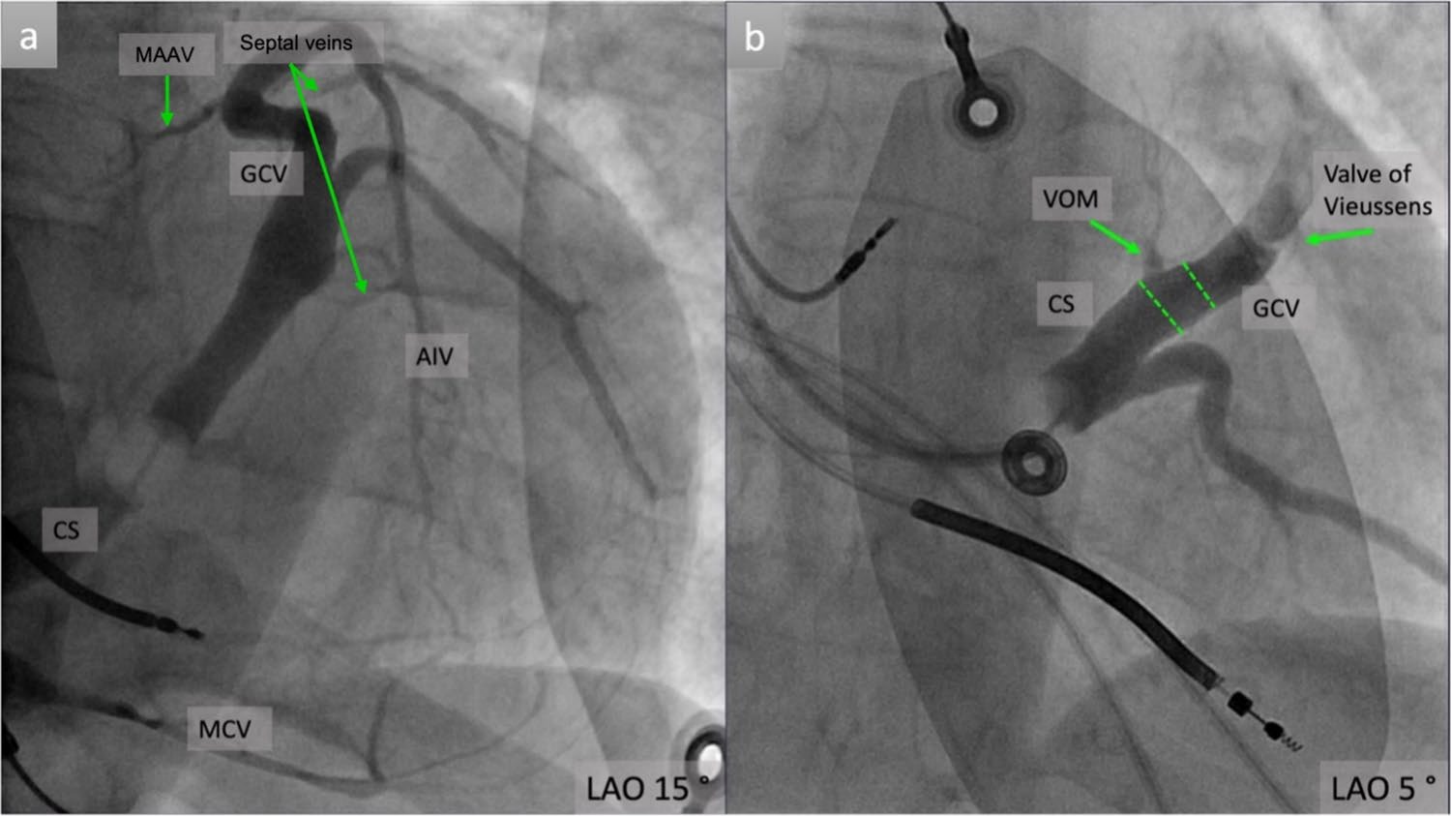
**a** Venous angiogram in the 15^o^ LAO projection showing the main tributaries of the coronary sinus; **b** The VOM in the 5^o^ LAO projection emerging from the CS-GCV junction; CS, coronary sinus; GCV, great cardiac vein; AIV, anterior interventricular vein; MCV, middle cardiac vein; VOM, vein of Marshall; MAAV, mitro-aortic annular vein; LAO, left anterior oblique projection

The main tributaries of the CS are the great cardiac vein (GCV), the anterior interventricular vein (AIV), the middle cardiac vein (MCV), the lateral and posterior veins of the LV and the vein on Marshall (VOM) (Fig. 1a).

## Coronary Sinus

The CS is the largest vein of the heart, running along the posterior left atrioventricular groove, spreading between the right atrium, where its ostium is provided with a fold (Thebesian valve), and the valve of Vieussens. Despite its constant presence, its dimensions are highly variable.

The diameter of the CS ostium varies between 5–15 mm and it is influenced by gender and the underlying heart disease [8–12]. The CS ostium tends to be larger in male patients, in the presence of significant mitral regurgitation and in patients with coronary artery disease. It is correlated with the LV size and has an inverse correlation with the LV ejection fraction [9, 13].

Regarding the length of the CS, it ranged between 15–70 mm in Ludinghausen's morphological study. In the angiographic study conducted by Simoon et al., the average length of the CS was 71.70 ± 9.71 mm, when the CS is formed from the GCV and left marginal vein (LMV), and 70.18 ± 14.98 mm when it is formed from the GCV and posterolateral vein [10, 11].

After the VOM take off from the CS slightly below level of the valve of Vieussens, the diameter of the main venous conduct of the heart becomes narrower and continues as the GCV [9] **(**Fig. 1b**).**

**Fig. 2:**
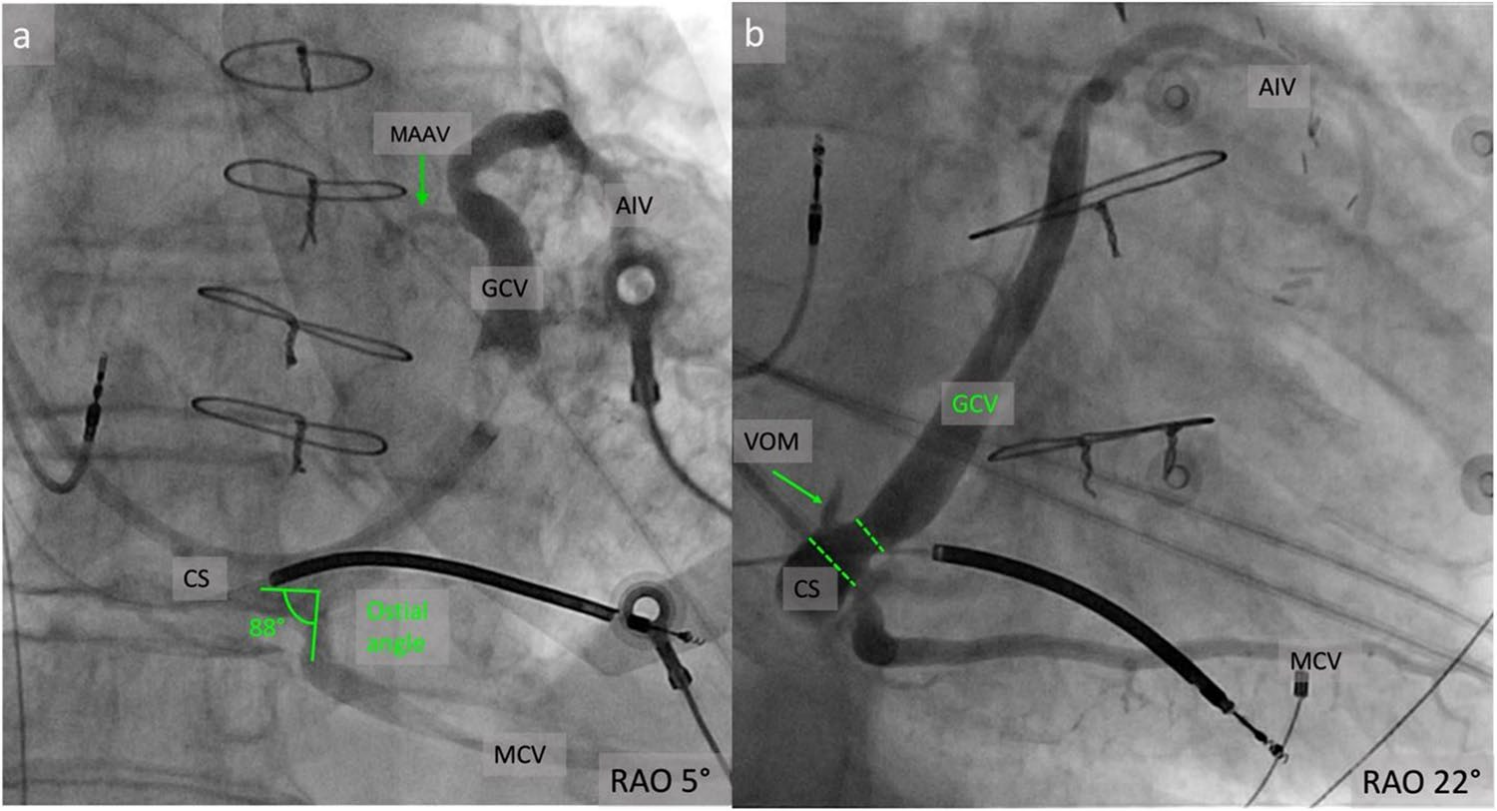
**a** A venogram showing an almost right ostial angle of the CS and **b** the origin of the GCV after the VOM take-off; CS, coronary sinus; GCV, great cardiac vein; AIV, anterior interventricular vein; MCV, middle cardiac vein; VOM, vein of Marshall; MAAV, mitro-aortic annular vein; RAO, right anterior oblique projection

The ostial angle of the CS, the angle between the CS ostium and the body of the CS, ranged from 65° to 151° with a mean angle of 119 ± 19°. It tends to be less than 90° in patients with tricuspid regurgitation because of the right atrial dilatation, which may cause difficulty in CS cannulation **(**Fig. 2a**)**.

In patients with severe mitral regurgitation and in patients with a coronary artery bypass graft (CABG), the CS tends to be displaced posteriorly and comes of the atrio-ventricular (AV) plane. In these cases, interventions performed from the CS, such as catheter ablation of an accessory pathway, require a strategy adapted to the altered anatomy. In addition, patients who have undergone a surgery for CABG tend to have significant changes in diameter along the main body of the CS (stenosis or aneurysm) [14].

Knowing the dimensions of CS before any procedure begins is helpful, especially when mitral isthmus ablation (MIA) is performed in patients with persistent atrial fibrillation [15]. In a study conducted by K. Wong Larger et al., involving 35 patients in whom MIA was performed, the CS diameter correlated significantly with total MIA (endocardial and epicardial CS ablation time) and CS ablation time. A CS diameter greater than 5.9 mm predicts the need for CS ablation [15].

CS cannulation is often difficult, resulting in longer CRT procedures and prolonged radiation exposure during fluoroscopy. The ostial diameter of the CS and its angle of opening into the right atrium, together with the anatomy of the Thebesian valve (TV) are elements responsible for successful cannulation of the CS [4].

The interest in exploring the anatomy of the TV is reflected in the published studies, in which different methods were used to visualize it, thus leading to several classification systems.

Mateusz K. Hołda et al. [16] conducted a study evaluating the dimensions and morphology of the TV via direct examination of autopsied human hearts. They measured the ostial diameter of the CS and the length and width of the TV and classified them according to their own classification system, creating 5 morphological types on the basis of the relationship between TV and the CS ostium. The results of the present study revealed that the transverse diameter of the CS was 12.2 ± 3.5 mm and the TV was present in 82.1% of the patients. They introduced a new idea that only the TV covering 100% of the CS ostium is considered obstructive, leading to difficulty in cannulation of the CS and these cases represent only a small percentage (2.6%) of all cases. Previous studies [17, 18] have shown that CS cannulation is unsuccessful in a percentage ranging between 2.87% to 3.7% of cases. By taking into consideration the two previous statements, the percentage of unsuccessful cannulations corresponds to a TV morphology that completely covers the CS ostium. This contrasts with the results of previous studies in which a valve covering more than 75% of the CS ostium was considered obstructive [19–21].

### Great Cardiac Vein

After the takeoff of the oblique vein of Marshall, the CS continues with the GCV **(**Fig. 2b**)**. It runs along the left atrioventricular groove, between the left atrium and left ventricle, near the circumflex artery. An interesting anatomical fact is that this is the only region in the human body where a vein and artery have the same direction of the blood flow. The valve of Vieussens marks the transition from the CS to GCV and it could cause difficulties when advancing the catheter and positioning the LV lead in CRT procedures. It is a structure present in about 60% of cases [19, 22] with variable morphology, with one to three leaflets that are concave/ flat, and are being inserted at the anterior (43.3%), antero-superior (16.7%), superior (15.6%), inferior (8.9%), posteroinferior or anteroinferior wall of the CS [22]. The ostial diameter of GCV was measured in two angiographic studies. The first one included patients with heart failure and the mean ostial diameter was 8.4 ± 1.8 mm [9], while the other one included patients with normal cardiac function. The mean ostial diameter of GCV was 6.34 mm ± 1.28 mm, being larger in males and in older patients [23].

### Anterior Interventricular Vein

The AIV, described in some works as a component of the GCV, originates at the apex, and runs into the anterior interventricular sulcus, where it is accompanied by the left anterior descending coronary artery. The junction between the GCV and the AIV usually has an ‘S-shape’ that passes through the superior, epicardial portion of the left ventricle, known as the left ventricular summit (LVS) region. The AIV receives some septal tributaries draining the anterior portion of the interventricular septum [12, 24]. AIV is currently use as an alternative pacing site in patients with tricuspid valve valvopathies or prosthetic tricuspid valve [25, 26]. It is also used for mapping and ablation of idiopathic ventricular arrythmias originating from the LVS [24, 27, 28].

### Middle Cardiac Vein

The MCV is also an integral part of the CS tree, as it is responsible for the venous drainage of the inferior cardiac wall. It flows along the posterior interventricular groove, originates at the apex and terminates near the CS **(**Fig. 1 and 2**)**. In an angiographic study of patients with heart failure, a mean diameter of 6.1 mm was determined [9]. Side branches may theoretically be utilized for lead placement, however these branches are generally quite apical and hence not suitable for lead placement [29].

### Left Marginal Vein

The LMV (obtuse marginal vein/ left lateral vein) drains the lateral part of the LV and opens into the GCV in 79% of cases or, in a smaller percentage of cases, into the CS. Depending on the method of evaluation, different percentages are given for the presence of the vein. According to angiographic studies, up to 91% of patients have a left marginal vein [9, 16, 30].

### Posterior and Lateral Veins of the LV

They are present in 50% of the cases. There is great variability in the number, tortuosity, and diameter of the posterior and lateral veins of the left ventricle. In biventricular pacing one of these veins is usually used for placement of the LV lead [6].

### Vein of Marshall and Other Left Atrial Veins

#### Vein of Marshall

The left atrial oblique vein, also known as the vein of Marshall, is an embryonic remnant of the left superior vena cava which is enclosed within the ligament of Marshall. During embryogenesis, the anterior cardinal vein obliterates, resulting in a fold that is the ligament of Marshall [31]. A detailed description of these structures dates back to 1850 when John Marshall, a cardiac surgeon, described the left atrial oblique vein as “remarkably constant structure” that frequently “measures from half an inch to an inch in length, and sometimes is as large as a crow-quill, but more commonly it is smaller, and will admit only the head of a pin” [32]. These anatomic structures that have been extensively described, in view of their importance in the pathogenesis of atrial fibrillation **(**Fig. 1**).**

Interest in understanding the mechanisms of atrial fibrillation, as well as in developing ablation techniques, led to several studies focusing on these structures [31]. The VOM is the main vein of the left atrium, draining the middle and lateral part of the posterior wall [7]. Different percentages of vein presence have been reported in the literature. It has reached 99% in necrotic studies [7, 33, 34] with a mean length of 2–3 cm and an average diameter of 1 mm. In a study based on multislice cardiac computed tomography scanning, 34% of the patients had the vein[35]. In studies based on venous angiographies, the percentage varied from 73% to 92.7% [31, 36]. This discrepancy might be explained by the fact that when the CS was cannulated and the occlusive balloon was inflated, in the research conducted by Kurotobi T et al., the orifice of the Marshall vein was also occluded.

Rodriguez-Manero M et al. described the angiographic anatomy of VOM in a population of patients with atrial fibrillation who underwent ethanol infusion as a complementary therapy to radiofrequency ablation [31]. They included 218 patients and VOM was cannulated in 86.2% of the patients; however, it was present in 92.7% of the patients, and was visualized by collateral flow [31]. The distance between the CS ostium and VOM ostium was 4.25 ± 2.57 cm. Regarding the morphology, most patients had a branching vein (78.2%), in 10.4% of cases, the VOM originated as a venous plexus, and in 12.2% of patients it was a stump without atrial branches [31]. Considering its anatomical relationships with the left pulmonary veins, the VOM is classified as follows: not reaching the left inferior pulmonary vein (LIPV) (17.6%), reaching the LIPV in most cases (72.8%) and passing over the left superior pulmonary vein [31]. In addition to postmortem studies, this method revealed the existence of many venous interconnections. The most common communication of the VOM is with the atrial appendage vein and with roof veins [31].

#### Left Atrial Veins

**Fig. 3:**
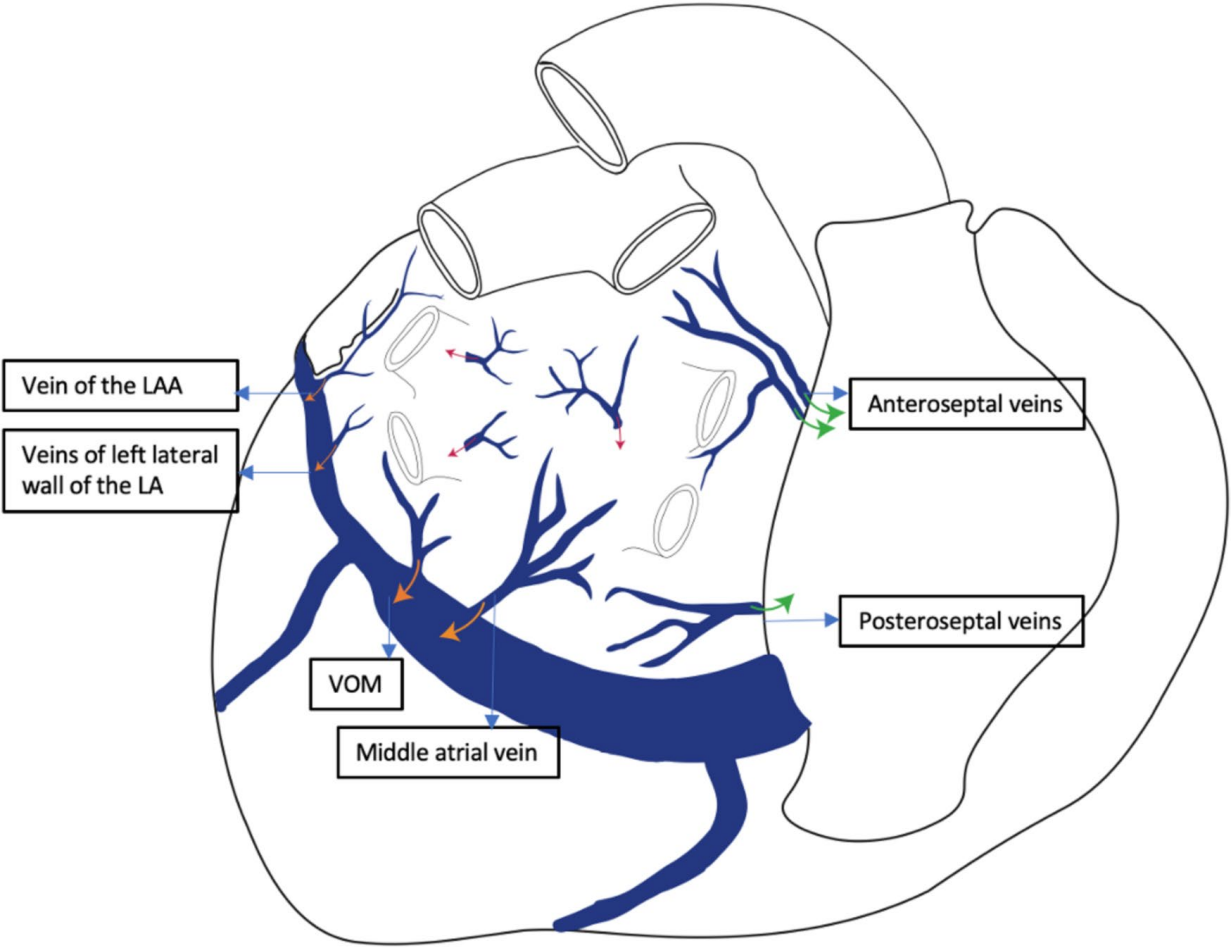
Venous drainage of the left atrium; VOM, vein of Marshall; LA, left atrium; LAA, left atrial appendage

Given the limited data available at this time on the distribution pattern of the left atrial (LA) veins, Von Ludinghausen was the first to describe venous drainage of the atrial myocardium. He identified the antero- and posteroseptal veins, posterior and oblique veins, lateral veins, auricular veins, and anterior veins and grouped them based on the territory that is drained, into: septal, posterior and posterosuperior veins **(**Fig. 3**).**

The septal veins were divided into two groups: anteroseptal and posteroseptal. The anteroseptal veins were described in 99% of the cases. They drain the anterior and septal walls of the left atrium, lie within the vicinity of the right pulmonary veins and open into the right atrium. In 78% of the heart specimens, these veins had valves. The posterolateral veins were described in only 34% of the specimens [33].

The posterior wall was drained by a middle vein in 78% of the cases and by a vein located in the left part of the posterior wall (VOM) in 99% of the patients. Both veins opened into the CS or GCV [33]. There are 2–3 veins small veins that drain the lateral wall of the LA and the left atrial appendage (LAA). All vessels emptied into the left coronary vein.

The last category is that of the so-called “proper veins” of the left atrium. It refers to several posterosuperior veins (one to three), described in 75% of cases, having a length of 2–3 cm, draining the region between the pulmonary veins and opening into the left atrium or into a pulmonary vein [33].

In addition to von Ludinghausen’s anatomic description, the angiographic view of the LA veins allows a better understanding of the distribution of vessels and the connections between them. In a study performed by Valderrabano et al., 218 CS venous angiograms were analyzed [31, 37].

LA veins opacification after injecting contrast into the CS allowed the description of atrial branches. The first vein to become visible, near the CS ostium is a so-called septal vein, which is detectable in 13% of cases, along the interatrial septum, in the lower part, drains into the right inferior pulmonary vein and is connected to other atrial veins: roof veins and posterior wall veins [37].

An inferior atrial vein was identified in 25.7% of patients, draining the inferior wall of the left atrium, as a branch of the oblique vein, as a branch of the septal vein or as a separate vein, which runs into the right inferior pulmonary vein or right superior pulmonary vein [37].

**Fig. 4:**
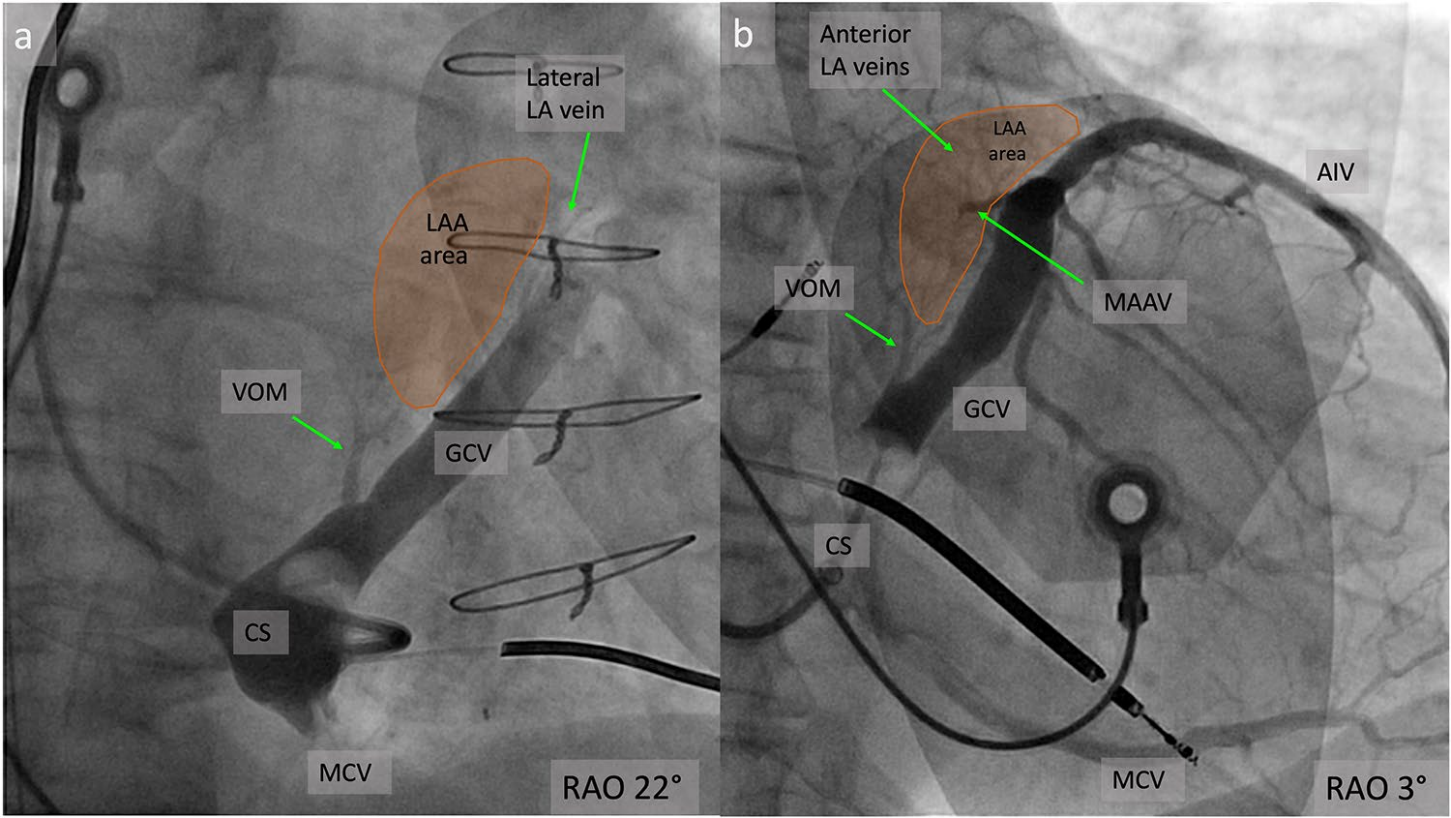
**a** RAO view of CS angiogram showing the VOM and the left atrial appendage vein; **b** Example of anterior left atrial veins having a collateral communication with a vein of the left ventricular summit ; CS, coronary sinus; GCV, great cardiac vein; AIV, anterior interventricular vein; MCV, middle cardiac vein; VOM, vein of Marshall; LA, left atrium; LAA, left atrial appendage; MAAV, mitro-aortic annular vein; RAO, right anterior oblique projection

The vein draining the LAA was identified in 53% of cases (Fig. 4a) [37].

Another venous branch, visualized in 19.3% of patients, is the anterior atrial vein, which arises distal to the LAA and collects blood from the LA roof **(**Fig. 4b**)** [37].

In addition to these veins, two Thebesian venous systems have been described: the first in the posterior wall of the LA and the second in the roof of the LAA [37]. The venous system draining the posterior wall of the LA was observed in 14.2% of angiograms after selective cannulation of a septal vein, an inferior vein or the VOM [37].

The second system consists of the veins of the LA roof, identified in 43% of cases, after selective cannulation of the VOM (35.8%), LAA (5%) or anterior vein (2.3%) [37].

### Left Ventricular Summit

A less described category of veins includes the veins of the LVS. There is very limited information available in the literature about the venous circulation in this area, from which a large proportion of idiopathic ventricular arrythmias originate [24, 27, 38]. The LVS is relatively difficult to access for ablation and the risks associated with this access are significant given the proximity of the left main coronary artery. Therefore, there is an increased interest in the coronary veins coursing in this area and could potentially provide a pathway to reach the LVS arrhythmogenic foci.

**Fig. 5:**
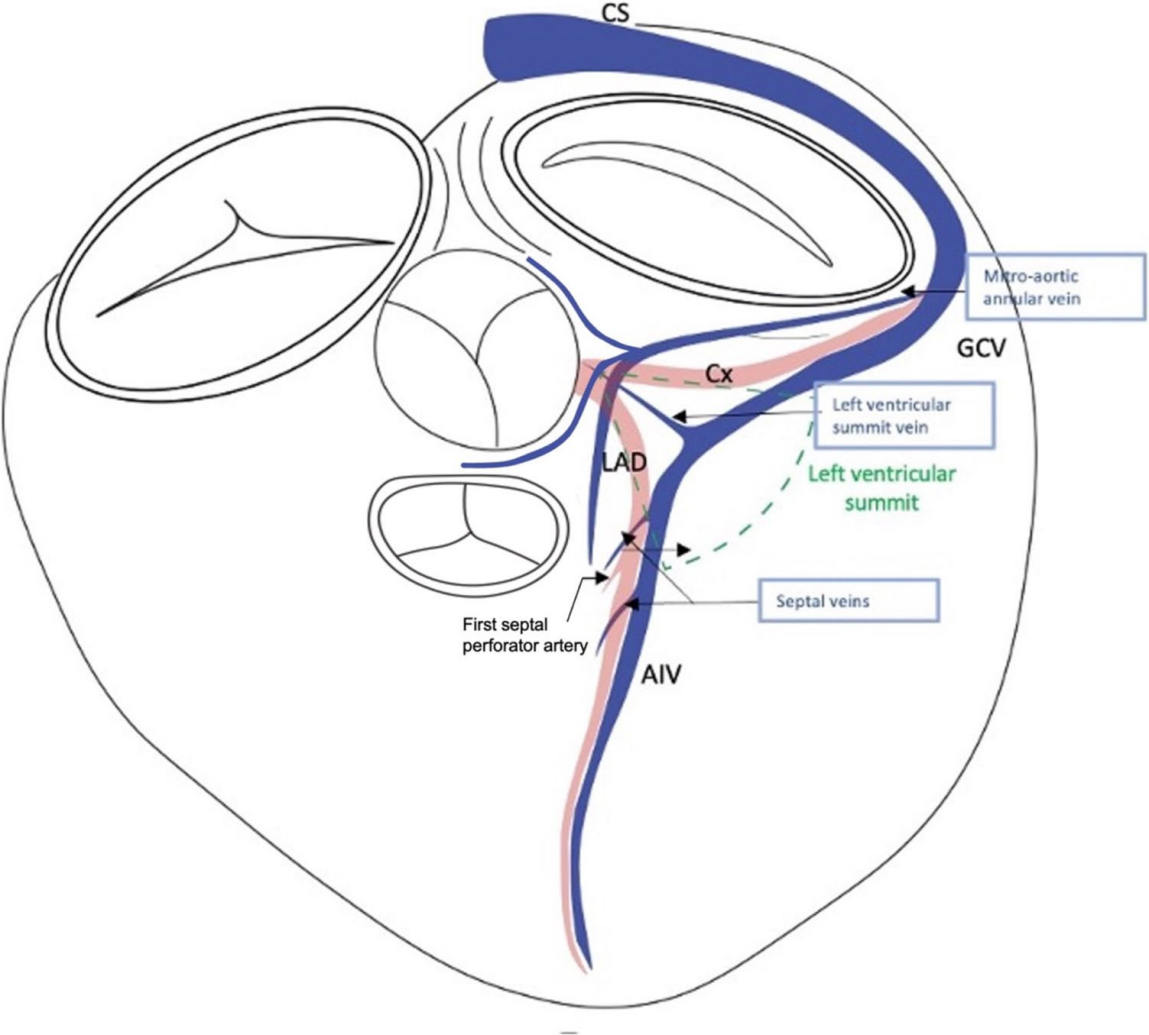
Venous anatomy of the left ventricular summit; CS, coronary sinus; GCV, great cardiac vein; AIV, anterior interventricular vein; Cx, circumflex artery; LAD, left anterior descending artery

The LVS represents a triangular area at the superior, epicardial region of the left ventricular outflow tract and is bounded by the left anterior descending artery (LAD), left circumflex artery (Cx) and an arched line that has the radius the distance from the left main bifurcation to the first septal perforator artery [39] **(**Fig. 5**).**

In addition to the scarcity of anatomical studies regarding the veins of the LVS, there is no consensus regarding their nomenclature.

We will divide these veins into two groups in order to better understand their distribution: veins that are in direct contact with the LVS, represented by the GCV-AIV junction and left ventricular summit vein (LVSV) and veins that travel close to it, including the mitro-aortic annular vein (MAAV) and septal veins.

### GCV- AIV Junction

The LVS is crossed by the junction between the GCV and the AIV. Successful mapping and ablation depend on the diameter, the transition angle between the veins and their anatomical relationship to the left main bifurcation.

Tavares et al. analyzed the GCV-AIV junction in 52 coronary computed tomographic angiographies. The transition angle was visualized and measured from two planes. From a lateral view, an obtuse angle (133 ± 14º) was present in 79% of cases and from a frontal view the transition between GCV and AIV was near the left main bifurcation at an almost right angle (88 ± 13 º) or more laterally with a less abrupt shift resulting in an obtuse angle (133 ± 12º) [24].

### Left Ventricular Summit Vein (LVSV)

The venous branch of the LVS originates from the GCV-AIV junction. Komatsu et al. named it ‘communicating vein’, while Tavares et al. refered to this vein as ‘first septal’ [24, 27]. According to their findings, this vein could be a true ‘perforator’, running deep into the septum, or it could turn towards the mitro-aortic continuity [24].

### Mitro-aortic Annular Vein (MAAV)

The MAAV is one of the veins that travels close to the LVS. This vein originates from the GCV and courses towards mitro-aortic continuity. After identifying it using retrograde venography, Komatsu et al. have canulated the ‘communicating vein’, as it was named, using a 2F microcatheter. The term ‘communicating vein’ is not defining the mitro-aortic annular vein, as it was also used for other branches that originate more distally, at the level of the GCV-AIV junction. [27].

A detailed angiographic description of the LVS veins was made by Tavares et. al. in a study that included 53 patients who underwent venous ethanol ablation for ventricular arrhythmias arising from the LVS [24]. The “left ventricular annular” (LVA) vein was observed in 36% of the patients, emerging from the GCV at the level of the mitral annulus and progressing toward the mitro-aortic continuity. Located at the border between the atria and ventricles, the catheter inside the vein detects both types of signals [24]. Ethanol infusion was successfully performed in 6 patient [24]. LVA is interconnected with the atrial veins, retroaortic branches and septal veins (58%) [24].

**Fig. 6:**
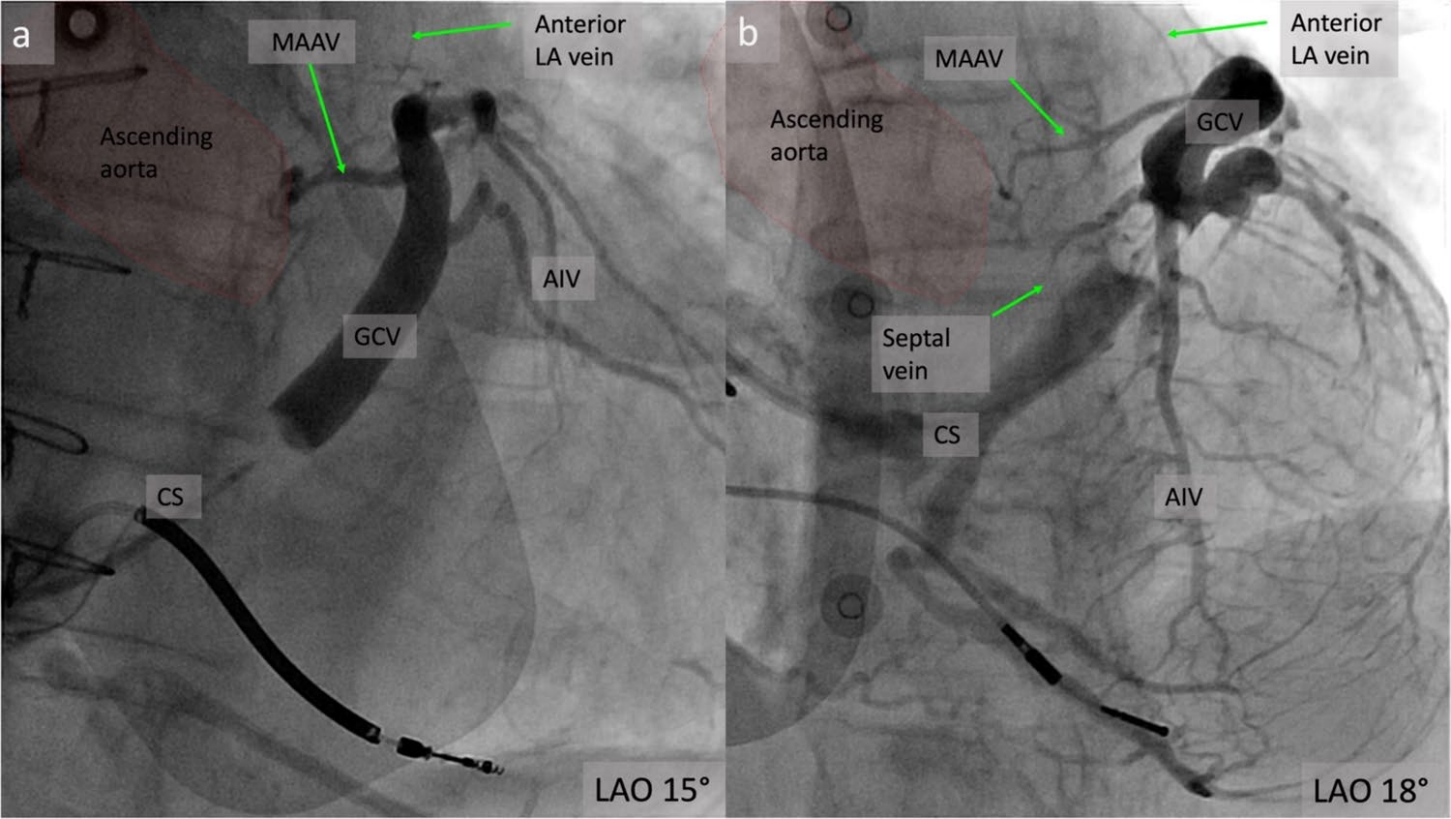
**a** CS angiogram showing the MAAV having collateral communication with anterior left atrial veins; **b** Septal veins emerging from AIV; CS, coronary sinus; GCV, great cardiac vein; AIV, anterior interventricular vein; LA, left atrium; MAAV, mitro-aortic annular vein; LAO, left anterior oblique projection

**Fig. 7:**
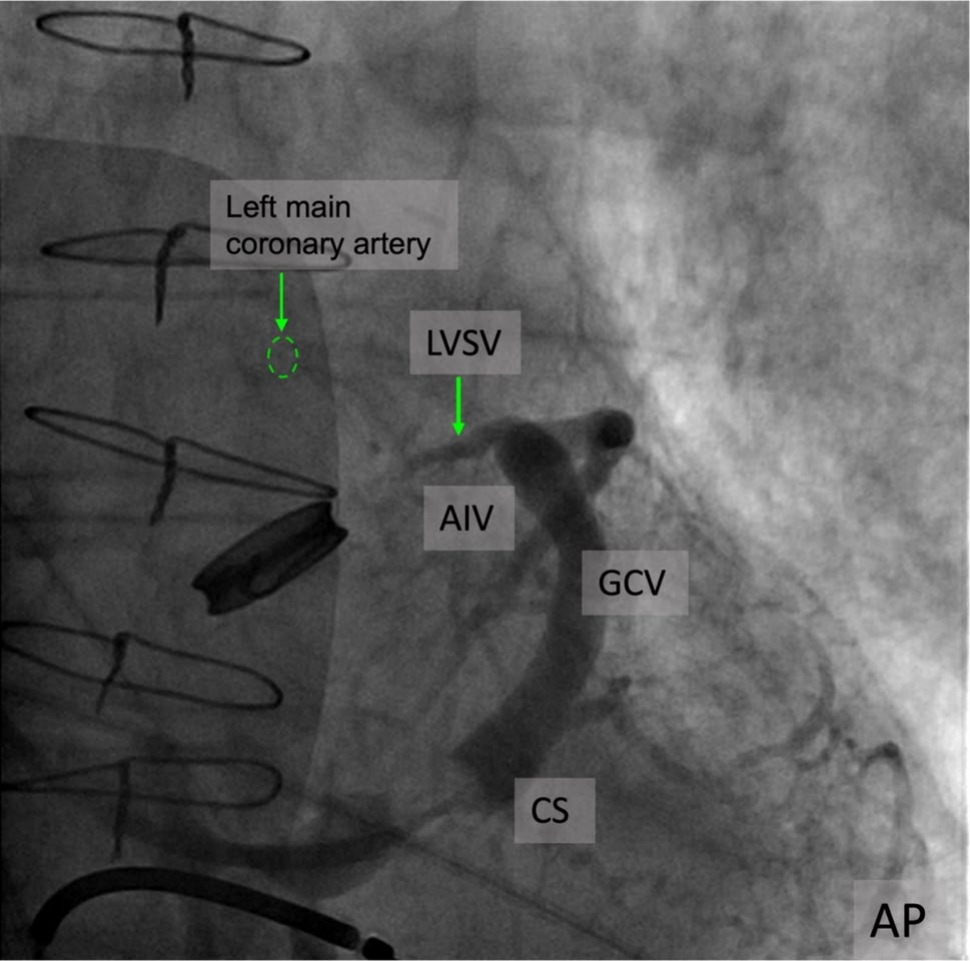
CS angiogram illustrating the trajectory of the LVSV in relationship with the aortic valve and the left main coronary artery; CS, coronary sinus; GCV, great cardiac vein; AIV, anterior interventricular vein; LVSV, left ventricular summit vein; AP, anteroposterior projection

In our series of patients undergoing CRT [38] we identified several veins in the LVS area. The MAAV emerged from the GCV, before GCV-AIV junction at 2 o’clock on the mitral ring in a left anterior oblique projection (LAO), coursed inside the left atrioventricular groove, towards the mitro-aortic continuity, where it turned towards the ventricular septum and coursed parallel to the AIV, tapered down and ended at midventricular level (Fig. 1a, Fig. 2a**, **Fig. 6). The LVSV emerged from the GCV, at the level of GCV-AIV junction at 12 o’clock on the mitral ring and then it had a similar course with the MAAV, running towards AMC and then turning towards the ventricular septum **(**Fig. 7**)**. In approximately three quarters of the angiograms, one of these veins was identified and there were patients having both. The total length of the MAAV was about 30 mm, of which 20 mm were inside the AV groove and 10 mm were along the ventricular septum. The length of the LVSV was about 20 mm. The length of the MAAV segment embedded in the left AV groove as well as the total length of MAAV correlated significantly with the LA diameter as well as with the right ventricular systolic pressure [38].

To summarize, LVSV and MAAV both originate at the level of GCV. The LVSV takes off at the junction between the GCV and AIV, aproximatelly at 12 o’clock when a CS angiogram is viewed in a LAO projection. The MAAV originates from the body of the GCV leftward from the LVSV takeoff, approximatelly at 1–2 o’clock in LAO projection. In most cases, both veins are oriented towards the AMC before making a turn along the ventricular septum. Branches of LVSV or MAAV can have a retroaortic path or run in between the aorta and the pulmonary artery **(**Fig. 5**).**

### Septal Veins

The septal veins are also described **(**Fig. 1a**, **Fig. 6b**)**. This group of veins consists of several intramural branches that originate in the interventricular septum and drain into the AIV at a 90-degree angle. From the origin of the AIV at the apex to its junction with the GCV, Tavares et al. identified one to four septal veins and all patients had at least one septal vein [24]. Most LVS septal veins are located anterior to the aortic root, posterior or anterior to the RVOT, in contrast to the LVA vein, which normally drains the posterior portion of the aortic root [24]. Ventricular arrhythmias were successfully ablated from the LVS septal veins in 38 patients: ethanol infusion was performed in 37 patients and radiofrequency ablation (RF) ablation was performed in one patient [24].

Recent research by Lijie Mi et al. has focused on the vein architecture in the LVS region [23]. They were able to characterize the veins anatomy, course, and size via high-speed rotational angiography. According to their definition, the LV summit veins are the veins draining near the GCV-AIV junction. They had identified three different types of veins based on the morphology and ostial diameter: type I “tiny branch”, type II “large trunk” and type III “venous network”. The most common type was type I (82%), which could permit venous ethanol ablation, whereas type II, in some cases, could fit a radiofrequency ablation catheter. Moreover, they classified the LVS veins into two groups, taking into consideration the relationship with the conduction system: “the upward drainage group” and “downward drainage group”. Excluding the “venous network” type, in 50% of cases, the summit veins collect blood form structures above the His bundle (aorta and RVOT or less commonly, the anterior RVOT, superior vena cava and right atrium). The other half of veins drain blood from structures at a lower level, closer to the His bundle, such as septal RVOT [23].

### Clinical Implications and Future Perspectives

The CV tree anatomy has practical importance in cardiac electrophysiology for more established procedures like lead placement for CRT and also for emerging techniques like vein of Marshall ethanol infusion during AF ablation and retrograde CV ethanol ablation for ventricular tachycardia substrates.

Ablation of arrhythmias originating from particular areas including the LVS, cardiac crux, perimitral area or interventricular septum can be attempted using techniques such as unipolar RF ablation, bipolar RF ablation and ethanol ablation [40, 41]. In case a CS ablation procedure is not possible because of the small caliber of the coronary veins, CS mapping with thin catheters could guide the endocardial approach. [42, 43]

Like any treatment involving vascular access, these procedures pose risks even though they offer the opportunity of treating the arrhythmia. Potential complications include coronary artery injury, vein dissection or perforation. A better understanding of the CS and its tributary veins distribution, dimensions and relationship with nearby structures could help the development of new ablation tools and strategies, with higher success rates.

Exploration of the septal veins seems to be promising, as their use for physiological biventricular pacing has recently been described. Min Soo Cho et al. proposed an alternative pacemaker lead trajectory for His-bundle pacing which involves implanting the lead into the septal perforator branch of the GCV[44]. This strategy is known as “cerclage parahisian septal pacing” [44]. A narrow QRS can be obtained from different parahisian sites, without direct His-bundle stimulation [45, 46]. The close relationship between the septal veins and the His bundle is also confirmed in a case report of a patient admitted for left bundle pacing [47]. As compared to conventional pacing strategies this technique eliminates tricuspid valve passage and its frequent complication: tricuspid regurgitation [44, 48].

The CV tree is more than just a passive conduit that facilitates other interventions. In an effort to better treat persistent atrial fibrillation, which is still a challenge, research on the involvement of the CS in the onset and maintenance of atrial fibrillation has been carried out [49]. Myocardial sleeves of CS wall have similarities to that of Purkinje fibers [50]. The significant presence of complex fractionated atrial electrograms (CFAEs) in the CS, together with the positive effects of CFAE ablation in persistent atrial fibrillation indicate that CS may play a part in the recurrence of atrial fibrillation after conventional ablation [49, 51–53].

## Conclusions

A better understanding of the CS and its tributary veins distribution, dimensions and relationship with nearby structures is important for optimization of outcomes in CRT as well as development of new ablation and pacing strategies.

## Key References

- Tavares L, Fuentes S, Lador A, Da-Wariboko A, Wang S, Schurmann PA, Dave AS, Valderrabano M. Venous Anatomy of the Left Ventricular Summit: Therapeutic Implications for Ethanol Infusion. Heart Rhythm. 2021. 10.1016/j.hrthm.2021.05.008
  - This study offers a detailed angiographic description of the left ventricular summit veins.
- Mi L, Zhang K, Zhang H, Ding L, Yu F, Weng S, Jiang Z, Zhang A, Dong X, Tang M. Venous anatomy of the left ventricular summit region: Insights from high-speed rotational retrograde angiography. J Cardiovasc Electrophysiol. 2023;34:2296–2304. 10.1111/jce.16064
  - This study describes the left ventricular summit veins via high-speed rotational angiography.

## Data Availability

No datasets were generated or analysed during the current study.
